# A giant spider from the Jurassic of China reveals greater diversity of the orbicularian stem group

**DOI:** 10.1007/s00114-013-1121-7

**Published:** 2013-12-07

**Authors:** Paul A. Selden, ChungKun Shih, Dong Ren

**Affiliations:** 1 College of Life Sciences, Capital Normal University, Beijing, 100048 China; 2 Paleontological Institute and Department of Geology, University of Kansas, Lawrence, KS 66045 USA; 3 Natural History Museum, London, SW7 5BD UK

**Keywords:** Araneae, Chelicerata, Deinopoidea, Hypochiloidea, Mesozoic, Orbiculariae

## Abstract

A large female spider, *Nephila jurassica*, was described from Middle Jurassic strata of north-east China and placed in the modern genus *Nephila* (family Nephilidae) on the basis of many morphological similarities, but, as with many ancient fossils, the single specimen lacked synapomorphies of the family (Selden et al. 2011). In order to test the placement within the nephilid phylogenetic tree, Kuntner et al. (2013) calibrated the molecular phylogeny using *N. jurassica* in three different scenarios based on inferred mitochondrial substitution rates. They concluded that *N. jurassica* fitted better as a stem orbicularian than a nephilid. Now, a giant male spider has been discovered at the same locality that yielded *N. jurassica*. The two sexes are considered conspecific based on their similar morphological features, size, and provenance. The male cannot be accommodated in Nephilidae because of its pedipalp morphology, so the new genus *Mongolarachne* and family Mongolarachnidae are erected for the species. Comparison with possibly related families show that Mongolarachnidae is most likely on the orbicularian stem, close to other cribellate orbicularians (e.g., Deinopoidea), which suggests a greater diversity of cribellate orbicularians during the Middle Jurassic.

**Electronic supplementary material:**

The online version of this article (doi:10.1007/s00114-013-1121-7) contains supplementary material, which is available to authorized users.

## Introduction

Palaeontology is littered with examples of fossils whose original interpretations have been overturned (in some cases literally) by the discovery of material which provides new evidence that was missing from earlier finds. Examples include the Cretaceous *Oviraptor* (Osborn, 1924), originally named for being an egg-stealer and later found to be a brooding theropod; *Hallucigenia* Conway Morris, 1977, described as a bizarre, stilting problematicum, was later shown to be an armoured onychophoran; and (closer to the taxon under discussion herein) *Attercopus fimbriunguis* (Shear, Selden and Rolfe, 1987), was first placed tentatively into the trigonotarbid arachnids (Shear et al. 1987), then redescribed as the oldest spider (Selden et al. 1991), and now forms the type of an extinct order of arachnids: Uraraneida Selden and Shear, 2008 (Selden et al. 2008). Normally, the new evidence comes many years later, but here we describe the male of a fossil spider species, first described only from the female, which came to light shortly after the original publication.

*Nephila jurassica* is a fossil spider which was described from a single, large, female specimen from the Jurassic of Daohugou, Inner Mongolia, China (Selden et al. 2011). It was placed in the modern genus Nephila on account of its large size and an assemblage of morphological features typical of that genus. The occurrence of a modern genus in strata as old as Jurassic seemed surprising, so Kuntner et al. (2013) tested three possible scenarios of calibration of molecular trees using this (and other) fossil data points. They concluded that *N. jurassica* is neither a *Nephila* nor a nephilid, but possibly a stem orbicularian. Here, we report the discovery of a giant male spider from the same locality, which we interpret as the male of *N. jurassica*. The distinctive palp, quite unlike modern nephilid palps, the plumose setal structure, and the presence of a calamistrum indicate that the species does not belong in Nephilidae, as predicted by Kuntner et al. (2013). We erect a new genus, *Mongolarachne* gen. nov., and new family, Mongolarachnidae fam. nov., to accommodate the species.

Comparison of the morphological features of *Mongolarachne jurassica* with potential modern relatives (Table 1) shows that certain features of the spider are found in the primitive hypochiloids, whilst others resemble those of cribellate orbicularians, such as the Deinopoidea. For example, study of both male and female specimens using a scanning electron microscope (SEM) reveals a plumose setal structure. It should be noted that that these are the first published SEM photographs of a non-amber fossil spider; the only previous publication of SEM photographs of a fossil spider was a preliminary report in 1976 of the use of SEM on a Baltic amber specimen (Mierzejewski 1976). We also submitted the type and only known specimen of the supposed araneoid *Juraraneus rasnitsyni* Eskov, 1984 from the Jurassic of Transbaikalia to SEM study. A recent restudy of this specimen showed that it is cribellate (Selden 2012), and the new SEM investigation reveals it has plumose setae, but different from those of *Mongolorachne* (see Electronic supplementary material Fig. 1). This is concordant with the results of Kuntner et al. (2013), who suggested it as a stem orbicularian.

**Table 1:** Comparison of *M. jurassica* ♀ and ♂ (Mongolarachnidae fam. nov.) with representatives of families with similar morphological features

		Mongolarachnidae		Orbiculariae							Haplogynae	Paleocribellatae			
		*Mongolarachne*			Araneoidea			Deinopoidea		Nicodamidae		Hypochilidae		Austrochilidae	
#	Character	♂	♀	*Juraraneus ♂*	*Nephila*	Araneidae	Tetragnathidae	Uloboridae	Deinopidae	*Megadictyna*	Filistatidae	*Hypochilus*	*Ectatosticta*	*Hickmania*	*Thaida*
1	Femoral trichobothria	0	0	0	0	0	1	1	0	0	0	0	0	0	0
2	Tibial trichobothria	0	0	1	0	1	1	1	1	3	2	3	3	1	1
3	Tibial gaiters	0	0	1	0	1	0	0	1	1	1	1	1	1	1
4	Third leg	0	0	1	0	0	0	0	0	1	1	1	1	1	1
5	Feathery setae	0	0	0	0	0	0	1	1	0	1	0	0	0	1
6	Plumose setae	0	0	0	1	1	1	0	0	0	0	0	0	0	0
7	Serrate setae	0	0	0	1	1	1	0	0	0	0	0	0	0	0
8	Serrate accessory claws	0	?	0	0	0	0	0	0	0	1	0	0	0	0
9	Tarsus 4 macrosetae	0	?	3	2	2	4	1	1	0	3	3	3	3	3
10	Tarsus 4 macrosetae shape	0	?	2	0	0	3	1	0	2	2	2	2	2	2
11	Calamistrum bristles	0	?	0	3	3	3	0	0	0	2	1	1	0	0
12	Calamistrum position	0	?	0	3	3	3	0	0	0	1	1	1	2	2
13	Calamistrum substructure	0	?	0	3	3	3	1	2	2	0	0	0	1	2
14	Male tarsi	0	–	0	0	0	0	0	0	0	1	1	1	0	0
15	Male palpal tibia	0	–	1	1	1	0	1	0	1	0	0	0	0	1
16	Male palpal tarsus	0	–	0	0	0	0	0	0	0	1	0	1	0	0
17	Male palpal embolus	0	–	2	2	2	1	2	1	1	0	0	0	2	2
	Score	17	7	10	9	7	7	9	11	10	6	9	8	9	7

Main references: Gray (1994), Griswold et al. (2005), Kuntner et al. (2008), Álvarez-Padilla and Hormiga (2011), Coddington et al. (2012)

We conclude that the new family Mongolarachnidae and the Juraraneidae are likely stem orbicularians and that there was a greater diversity of cribellate orbicularians in the Middle Jurassic than today.

## Materials and methods

The specimens come from finely laminated, pale grey tuff near Daohugou Village, Wuhua Township, Ningcheng County, Inner Mongolia, China (41°19.532′ N, 119°14.589′ E). The Daohugou beds were deposited in lacustrine conditions in a volcanic region (Ren et al. 2002) and are well known as a Fossil–Lagerstätte bearing plants, insects, crustaceans, arachnids, molluscs, amphibians, reptiles, and mammals. A Middle Jurassic age for the Daohugou biota has been proposed based on the composition of the insect fauna (Ren et al. 2002; Huang et al. 2006), conchostracans (Shen et al. 2003), and isotopic dating (Chen et al. 2004; Liu et al. 2004). The material is deposited in the College of Life Sciences, Capital Normal University, Beijing.

**Fig. 1:**
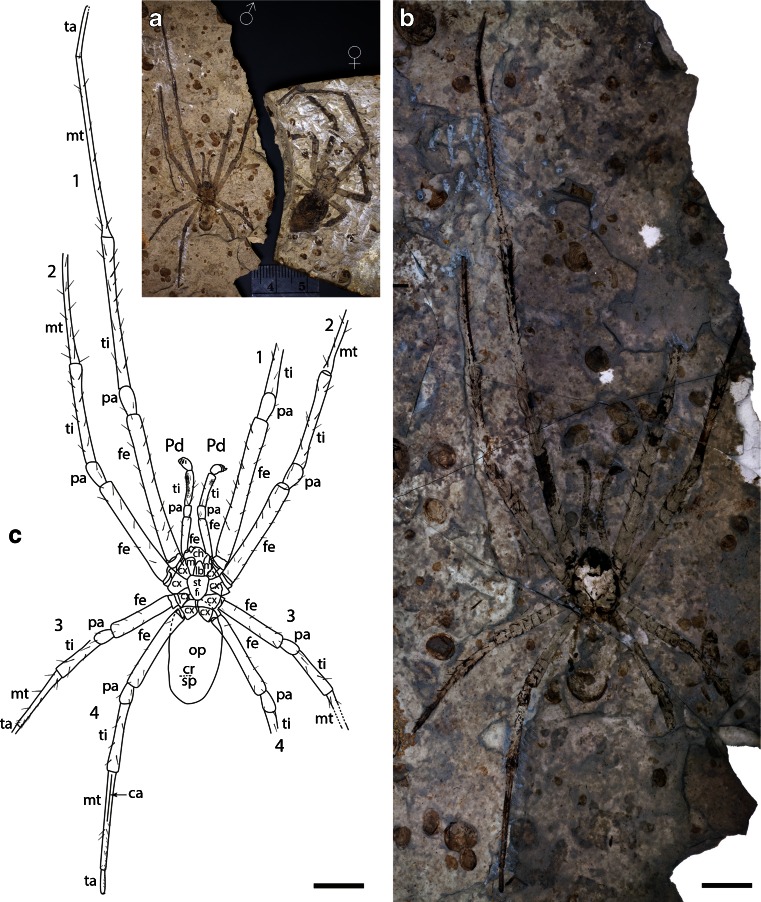
*M. jurassica*: **a** allotopotype male CNU-ARA-NN2011001-1 (♂) and holotype female CNU-ARA-NN2010008 (♀) specimens compared; **b** allotopotype male part; for explanation see (**c**). **c** Allotopotype male part, explanatory drawing of (**b**). Abbreviations: *1, 2, 3, 4*, leg numbers; *ca*, calamistrum; *ch*, chelicera; *cr*, cribellar area; *cx*, coxa; *f*, fovea; *fe*, femur; *lb*, labium; *mt*, metatarsus; *mx*, maxilla; *op*, opisthosoma; *pa*, patella; *Pd*, pedipalp; *st*, sternum; *ta*, tarsus; *ti*, tibia; *tr*, trochanter. Photographs **a** dry, **b** under polarized light with specimen under 70 % ethanol; scale *bars* = 5 mm

**Fig. 2:**
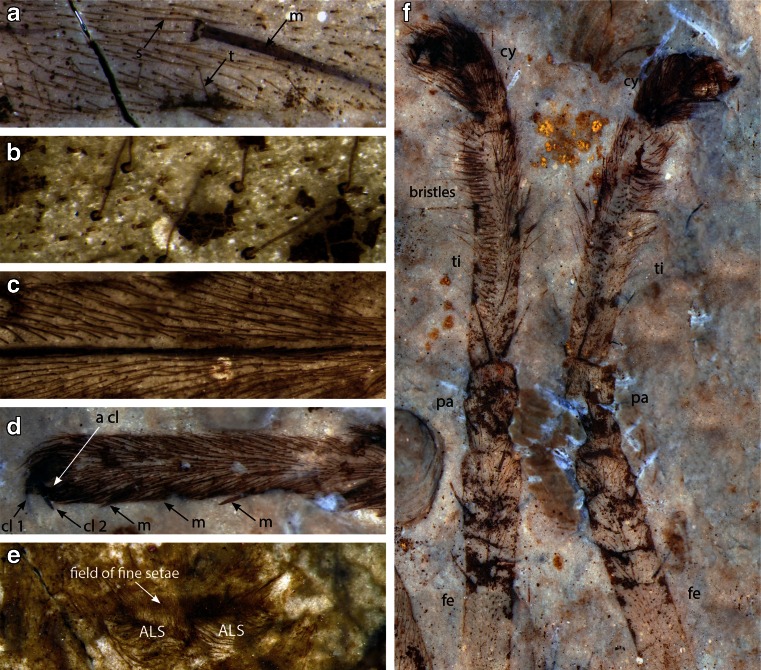
*M. jurassica*, allotopotype male part CNU-ARA-NN2011001-1 (except **e**: counterpart CNU-ARA-NN2011001-2), morphological details; photomicrographs taken in polarized light with specimen under 70 % ethanol: **a** Right leg 1 tibia showing cuticular structures: *m*, macroseta; *s*, seta; *t*, trichobothrium; *b* higher magnification of trichobothria of left leg 4, showing crescentic bothrial base; distal to the right; **c** basal part of left leg 4 metatarsus showing detail of calamistrum and simple setae; distal to the left; **d** tarsus of left leg 4, showing one of the paired claws (cl 1), another claw (cl 2) which could be the median claw or the second paired claw, accessory claws (S-shaped serrated setae, one shown at a cl), and row of distinctive, sustentaculum-like macrosetae (*m*); distal to the left; **e** spinneret region of counterpart specimen, showing wide, oval field of fine setae anterior to anterior lateral spinnerets (*ALS*); **f** pedipalps showing elongated tibiae with longitudinal field of bristles

**Fig. 3:**
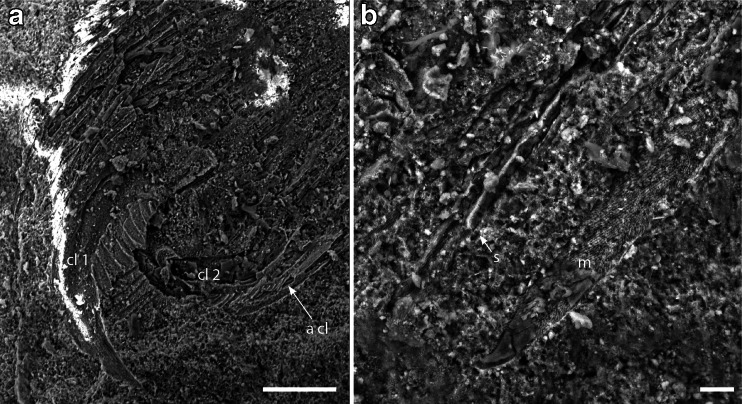
*M. jurassica*, allotopotype male part CNU-ARA-NN2011001-1, SEM photographs: a tip of tarsus 4 (compare with Fig. 2d); paired claw (cl 1) showing seven blade-like pectines, possible median claw (cl 2), and serrate accessory claw (**a** cl); scale *bar* = 100 μm; **b** distal part of macroseta of tarsus 4 (left macroseta in (Fig. 2d)) showing curved tip, and numerous setae (one shown at *s*); note that both macroseta and setae have an infill of smooth, translucent (crystalline?) material (*below m*, extending to tip; *above s arrow*), but where this is broken away, the external surface (*above m; left of s arrow*) shows a distinctive linear or spiral pattern of short barbs (cf. Lehtinen 1967, Fig. 8); scale *bar* = 20 μm

The specimens were studied and photographed under 70 % ethanol using a Leica MZ16 stereomicroscope, and photographed using a Canon EOS 5D MkIII digital camera attached to the microscope and DSLR Assistant software (www.kaasoft.com) on an Apple MacBook Pro computer (Figs. 1a, b; 2a–f; and 3a). Figure 2, panels g and h, were made on a Leo 1550 field-emission SEM; see Electronic supplementary material for details. Figure 3c was photographed on a Zeiss Sigma Variable Pressure Field Emission SEM. These are the first SEM photographs to be published of matrix-preserved fossil spiders (Selden and Penney 2010). Photographs were manipulated using Adobe Photoshop software, and final drawings were made from the photographs using Adobe Illustrator. All measurements are in millimeters and were made from the photographs using Photoshop’s analysis tool.

Table 1 is a comparison chart of various characters visible on the fossils with those of possible relatives. The modern comparators were chosen because they show morphological similarities to the fossils. The morphological characters are discussed below, with their states as shown in Table 1 (coded as 0 for their state in *Mongolarachne*, ? = not known in the fossil, all unordered). The matrix in Table 1 was analyzed using Mesquite 2.75 (http://www.mesquiteproject.org) (for which *Mongolarachne* male and female were merged) as an aid to examining the possible relationships of the Mongolarachnidae).

## Morphological characters

Femoral trichobothria are absent in all taxa compared, including *Mongolarachne*, except that they occur on one or more femora in uloborids (Griswold et al. 2005; Opell 1979) and many tetragnathids (Álvarez-Padilla and Hormiga 2011). All taxa have short tibial trichobothria, but only in *Mongolarachne* and Nephilidae do these form a large cluster; in others, they are in one or two rows. Femoral trichobothria: 0 absent, 1 present; tibial trichobothria: 0 cluster, 1 present, 2 one row, 3 two rows.

Brushes of long setae on the ends of the tibiae (gaiters) occur only in female Nephilidae (legs 1, 2, and 4), *Mongolarachne* (all legs), *Uloborus* (leg 1), and the tetragnathid *Opadometa* (leg 4) (Table 1), although setal brushes also occur on leg 1 of many male spiders for signalling to mates (e.g., Framenau and Hebets 2007, Miller et al. 2012). Tibial gaiters: 0 present, 1 absent.

The third leg is very short (<0.5× length of leg 1) in Orbiculariae and in *Mongolarachne*; in other web spiders, it is also usually the shortest, but less so (>0.5× length of leg 1) (measurements from specimens and the following literature: Kraus 1956; Gertsch 1958; Hoffman 1963; Catley 1994; Gray 1994; Harvey 1995; Ramírez and Grismado 1997; Opell 1983; Yin et al. 2002; Penney 2003; Chang and Tso 2004; Joseph and Framenau 2012). Third leg: 0 short, 1 long.

Definitions of cuticular hair types follow Lehtinen (1967, Figs. 8–10) and Griswold et al. (2005, Figs. 147–148) (contra Comstock 1912; Green 1970; Cushing 2005), viz.: plumose setae are normal body setae which bear abundant fine projections, generally in lines or whorls, over their entire surface; in some spiders, these are coarse and visibly different from simple setae with the naked eye (e.g., Dictynidae) while, in others, the fine projections are visible only under very high magnification (e.g., SEM). The latter is the kind of plumose seta found in the fossil specimens. Feathery setae are dendritic, with lateral branches resembling the veins in a leaf, which normally lie flat on the cuticle surface (e.g., Agelenidae: Bolzern et al. 2013, Fig. 2a). Simple and serrate setae are normal, fine, body setae found in Araneoidea; the former are smooth while the latter bear sparse, minute projections (Griswold et al. 2005). Plumose setae are present in all taxa compared except araneoids. Feathery setae have not been observed in *Mongolarachne*, despite extensive searches in both light and scanning electron microscopy. Feathery setae: 0 absent, 1 present; plumose setae: 0 present, 1 absent; serrate setae: 0 absent, 1 present.

Some spiders bear serrate accessory setae (=serrated bristles or false claws) adjacent to the median tarsal claw; these appear as gently s-shaped macrosetae (from which they are presumably derived) with ventral thorns. They function in conjunction with the median claw in manipulating silk on the web (Foelix 1970) and are characteristic of web-living spiders. In their Atlas of Entelegynae, Griswold et al. (2005) distinguished between these and sinuous plumose setae, seen, for example, in *Phyxelida* and *Filistata* (Griswold et al. 2005, Figs. 132C and 136C, respectively) which presumably have a similar function. However, they scored the Hypochilidae as lacking serrate accessory setae, yet they do occur in both *Hypochilus* and *Ectatosticta* (Electronic supplementary material, Fig. 3a–d, f). The serrate accessory claws of *Mongolarachne* (Figs. 2d and 3a) resemble those of *Deinopis* (Griswold et al. 2005, Fig. 135E). Serrate accessory claws: 0 present, 1 absent.

The sustentaculum is a distinctive macroseta on the ventral side of the distal end of tarsus 4 adjacent to the serrated bristles in Araneidae (Scharff and Coddington 1997; Griswold et al. 1998; Álvarez-Padilla and Hormiga 2011), and a line of such macrosetae on the fourth metatarsus and tarsus has also been mentioned for some araneid genera (Álvarez-Padilla and Hormiga 2011). Supposed sustentaculum-like macrosetae have been described in Nephilidae (Kuntner 2005, 2006) and Synotaxidae (Agnarsson 2003), but in these cases the shape of the macroseta is quite different from those of araneids, although it is in the same position on the tarsus. A row of sustentaculum-like macrosetae occurs on metatarsus–tarsus 4 of *Mongolarachne*, which is similar to the comb of similarly shaped macrosetae in Deinopidae (Griswold et al. 2005, Fig. 141B; Coddington et al. 2012, Fig. 5f), although these macrosetae are weakly organized in *Menneus* (Griswold et al. 2005). In contrast, the macrosetae in the uloborid comb are short and sculptured (Opell 1979, plate 1A, C). In Nicodamidae, *Megadictyna* has sustentaculum-like macrosetae uniformly distributed, while in *Nicodamus* similar macrosetae are not arranged in a comb (Griswold et al. 2005). Some other araneoids have macrosetae on the fourth tarsus, but these are not sustentaculum-like (Griswold et al. 2005). Among the taxa bearing sustentaculum-like macrosetae, there seems to be a variation from those arranged in a loose row (*Mongolarachne*, *Nicodamus*), through those in a distinct comb (*Megadictyna*, *Deinopis*), to the single sustentaculum (araneids, nephilids), which is likely derived with respect to the others (Griswold et al. 2005; Kuntner et al. 2008). The reduction of the row or comb could be related to the loss of the cribellum and calamistrum. Tarsus 4 macrosetae: 0 row, 1 comb, 2 sustentaculum, 3 one or more, 4 absent; tarsus 4 macrosetae shape: 0 sustentaculum-like, 1 sculptured, 2 simple, 3 absent.

The calamistrum varies in number of rows of bristles, length, position on the metatarsus, and whether is it situated on a pinched ridge or a shallow depression. The calamistrum is situated in an excavation of the metatarsus in Uloboridae (Opell 2001, Fig. 2; Griswold et al. 2005, Figs. 142E and 145A) and adjacent to an excavation in *Hickmania* (Gertsch 1958, Fig. 44); it is on a ridge in Deinopidae (Peters 1992, Fig. 8d) and *Megadictyna*, and adjacent to a ridge in *Thaida* (Griswold et al. 2005, Fig. 144A) (also pers. obs. for all). Calamistrum bristles: 0 uniseriate, 1 biseriate, 2 triseriate, 3 absent; calamistrum position: 0 proximal ½, 1 proximal ¼, 2 medial, 3 absent; calamistrum substructure: 0 none, 1 excavation, 2 ridge, 3 absent. The associated cribellum is poorly preserved, but the spinneret region is visible in the counterpart (Fig. 2e, Electronic supplementary material Fig. 2c). A wide, oval field of fine setae in front of the anterior lateral spinnerets (which are both rotated to the right) resembles a similar patch of fine setae in some other cribellates (e.g., Griswold et al. 2005, Fig. 103; Davies 1993, Figs. 2 and 3) and may represent a vestigial cribellar region.

The tarsi of males of Filistatidae are slightly sinuous (Electronic supplementary material Fig. 3j), and those of hypochilid males are curved (Electronic supplementary material Fig. 3a,d; Doran et al. 2001, Fig. 1d). Male tarsi: 0 straight, 1 curved or sinuous.

The elongate tibia of the male pedipalp of *Mongolarachne* is unusual in spiders but is similar to those of the primitive araneomorph family Hypochilidae (Forster et al. 1987; Lehtinen 1967, Figs. 14–16), some Tetragnathidae (Álvarez-Padilla and Hormiga 2011), and the filistatids *Filistata* and *Kukulcania*, for example. However, in *Ectatosticta*, the tarsus is also greatly elongated and bears distinctive spines, whereas the pedipalp tarsus in *Mongolarachne* is very short, and the tibia of *Ectatosticta* does not bear the distinctive bristles seen in *Mongolarachne* (Fig. 2f). Elongation of the male pedipalp of the filistatid *Kukulcania* is mainly achieved by a long femur and tibia, as in *Mongolarachne* (Barrantes and Ramírez 2013). Elongate pedipalps occur sporadically in spider families outside of the comparators listed here, for example, in the mimetid *Gelanor* (Shear 1981), for individual functional reasons. The tarsus is a spoon-shaped cymbium in most spiders; even if elongated (as in Austrochilidae), it is not much longer than the sclerites which it accommodates and which are attached laterally. In Filistatidae and Ectatosticta, the palpal sclerites are located at the tip of an elongated tarsus. The embolus of *Mongolarachne* is spiral and very similar to that seen in *Ectatosticta* (Forster et al. 1987, Fig. 78). The embolus is elongate in *Nephila* and weakly spiral or planispiral in Tetragnathidae. The pedipalp organ in *Deinopis* is distinct, with tight planispiral coiling (Coddington et al. 2012). Male palpal tibia: 0 elongate, 1 short; male palpal tarsus: 0 cymbium, 1 long; male palpal embolus: 0 spiral, 1 planispiral, 2 not spiral.

## Systematic palaeontology

Order Araneae Clerck, 1757

Suborder Opisthothelae Pocock, 1892

Infraorder Araneomorphae Smith, 1902

Family Mongolarachnidae fam. nov.

Etymology from the genus *Mongolarachne* gen. nov.

### *Diagnosis*

A family of araneomorph spiders distinguished by the following combination of characters: both sexes of large size; cribellate, with straight, uniseriate calamistrum occupying proximal half of fourth metatarsus; distal metatarsus and tarsus of fourth leg with row of sustentaculum-like macrosetae; finely plumose setae; lacking feathery setae; cluster of many trichobothria in proximal half of all tibiae.

This family differs from Juraraneidae by the structure of the male palp; from Recent Araneoidea by the presence of plumose setae and a calamistrum in Mongolarachnidae; from Deinopoidea by the tibial trichobothria in a large cluster, lack of feathery setae, and the shape of the male palpal embolus in Mongolarachnidae; from Nicodamidae by the tibial trichobothria in a cluster, presence of tibial gaiters, short third leg, curved tip to tarsus 4 macrosetae, and male palpal structure in Mongolarachnidae; from Filistatidae by a cluster of tibial trichobothria, tibial gaiters, short third leg, lack of feathery setae, presence of serrate accessory claws, tarsus 4 macrosetae, calamistrum structure, and straight tarsi and palp structure in the male of Mongolarachnidae; and from Palaeocribellatae by the cluster of tibial trichobothria, tibial gaiters, short third leg, tarsus 4 macrosetae, and calamistrum position in Mongolarachnidae (see Table 1).

Type genus *Mongolarachne* gen. nov. The family is monotypic.

Etymology from the Inner Mongolia Autonomous Region, where the fossils were discovered, and the Greek άράχνη (L. arachne), a spider.

Diagnosis: Tibial gaiters on leg 3 as well as on all other legs (weak in the adult male); male pedipalp extremely elongate, with especially long tibia bearing field of bristles along its length; female epigyne nose-shaped.

**Fig. 4:**
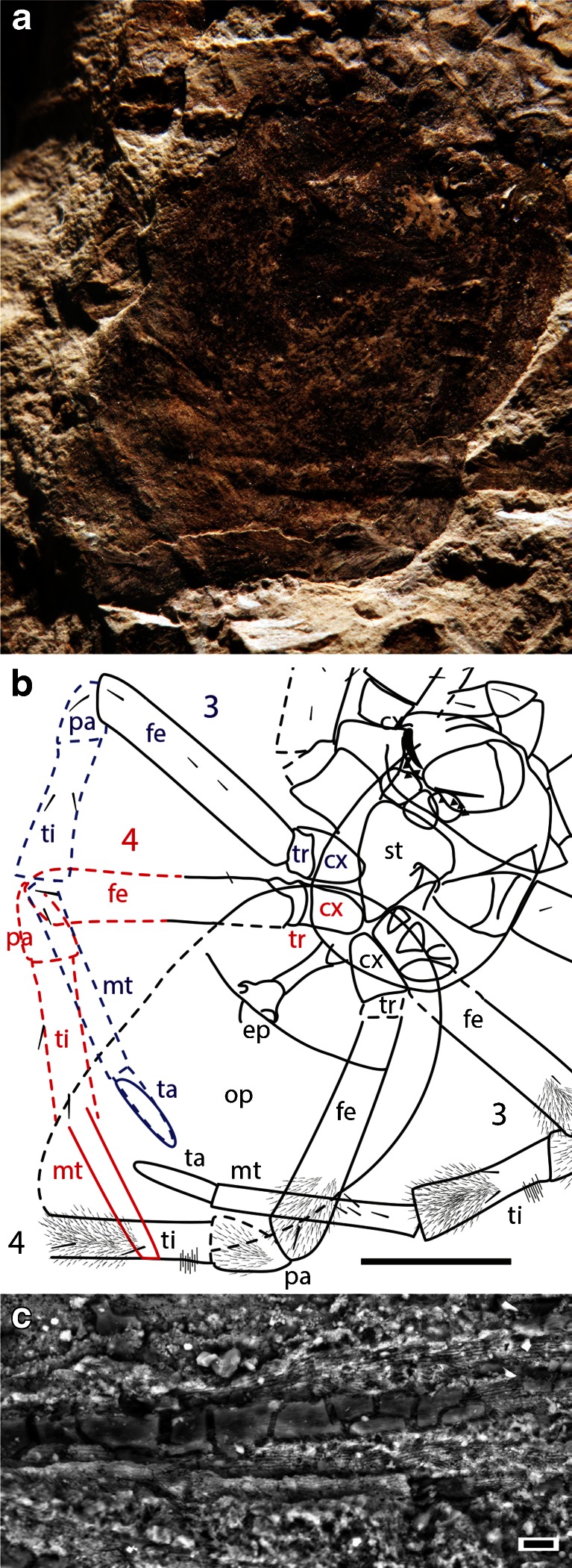
*M. jurassica*, holotype female CNU-ARA-NN2010008 opisthosoma and posterior legs: **a** photograph in low-angle light of dry specimen; see **b** for explanation; **b** explanatory drawing of **a**; *3, 4*, leg numbers; *cx*, coxa; *fe*, femur; *mt*, metatarsus; *op*, opisthosoma; *pa*, patella; *st*, sternum; *ta*, tarsus; *ti*, tibia; *tr*, trochanter; *blue* and *red* show left legs 3 and 4, respectively, mirrored from preserved right legs; *dashed lines* are inferred morphology; scale *bar* = 5 mm; **c** SEM photograph of numerous leg setae, showing infill of smooth, translucent (crystalline?) material, where broken away reveals external pattern of short barbs, as in the male; scale *bar* = 10 μm

Type species *N. jurassica* Selden, Shih and Ren, 2011 (Figs. 1, 2, 3, and 4; Electronic supplementary material Fig. 2).

*M. jurassica* (Selden, Shih and Ren, 2011) comb. nov.

*Diagnosis*: As for the genus.

*Holotype*: A female CNU-ARA-NN2010008.

*Other material*: An allotopotype male CNU-ARA-NN2011001-1 (part) and CNU-ARA-NN20110001-2 (counterpart). Both specimens from the finely laminated, pale grey tuff near Daohugou Village, Wuhua Township, Ningcheng County, Inner Mongolia, China (41°19.532′ N, 119°14.589′ E).

### *Description of allotopotype male*

Adult male: cuticular structures: setae and macrosetae finely plumose (Fig. 2g, h), no feathery setae (Fig. 2a); short trichobothria in large cluster (>30, fewer on Pd) in basal half of all tibiae, bothria asymmetric: thicker, crescentic rim proximally which thins distally (Fig. 2b); finely plumose macrosetae (Fig. 2a) on all post-trochanteral podomeres, many and large on dorsal surfaces, except femora, where shorter, thinner, sparse on ventral surfaces, except metatarsus 3 which has dense macrosetation in distal half.

Carapace pyriform, curved lateral sides, tapering forwards, more rounded posterolaterally. Eyes poorly preserved. Labium tongue-shaped, straight posterior border, lateral sides curve to meet anteriorly, longer than wide. Sternum shield-shaped, scalloped laterally, pointed posteriorly but not produced between coxae 4. Chelicera short, not porrect, subtriangular in lateral aspect. Pedipalp with elongated femur and tibia (Fig. 2f); latter bearing conspicuous longitudinal field of bristles (short macrosetae), c. 3-wide, running from subproximal–ventral to lateral–distal; cymbium suboval, densely setose, especially ventrally and distally, with setae covering more distal palpal elements; other parts of pedipalp incompletely preserved, but suggestion of spiral element proximally, distal parts of embolus not preserved. Legs long, slender; leg formula 1243; weak gaiters on distal ends of tibiae 1–4; tarsus 4 (Fig. 2d) with 3 claws and accessory claws; distal ½ of metatarsus and tarsus 4 with row of asymmetric macrosetae with slightly curved tips (resembling sustentacula) (Fig. 3b) ventrally; proximal ½ of metatarsus 4 with calamistrum composed of straight, single row of bristles dorsally (Fig. 2c). Opisthosoma cylindrical with rounded anterior and posterior. Wide, oval field of fine setae (Fig. 2e; Electronic supplementary material Fig. 2c) situated c. 2/3 of length of opisthosoma from anterior, anterior lateral spinnerets posterior to this field, smaller posterior lateral spinnerets.

Measurements (millimeters): body length 16.54; carapace length 6.74, width 5.05; opisthosoma length 10.86, width 5.42, length/width ratio 2.00; cribellar area width 1.97; sternum length 2.55, width 2.31, length/width ratio 1.10; labium length 1.58, W 1.18, length/width ratio 1.34; maxilla length 1.77, width 0.94, length/width ratio 1.88; leg formula (longest to shortest): 1243; podomere lengths: pedipalp femur 4.05, patella 1.24, tibia 3.79, tarsus 1.68, total (fe–ti) 9.08, total (fe–ta) 10.76; leg 1 coxa 1.74, trochanter 0.85, femur 15.10, patella 2.99, tibia 15.96, metatarsus 19.00, tarsus ≥5.35, total (fe–ti) 34.05, total (fe–ta) ≥58.16; leg 2 coxa 1.84, trochanter 0.69, femur 11.46 patella 2.67, tibia 10.43, metatarsus >11.53, total (fe–ti) 24.56; leg 3 coxa 1.69, trochanter 0.54, femur 6.76, patella 1.99, tibia 5.56, metatarsus 6.97, total (fe–ti) 14.31; leg 4 coxa 1.83, trochanter 0.57, femur 9.13, patella 2.21, tibia 7.39, metatarsus 10.38, tarsus 2.64, total (fe–ti) 18.73, total (fe–ta) 31.68.

### *Additions to interpretation of holotype female*

The original description (Selden et al. 2011) described the setae as simple, not plumose, which is how they appear under the light microscope. In this investigation, the specimen was studied under the SEM, which shows that the setae are, in fact, finely plumose (Fig. 4c).

The opisthosoma shows additional details when viewed under low-angle incident light (Fig. 4a, b). Along the midline about halfway between the opisthosoma and the posterior tip of the opisthosoma lies a wide, short, elliptical structure (width 2.95). Posterior to this structure, about halfway between it and the posterior end of the opisthosoma, lies a transverse ridge. Right leg 3 is seen to continue as a similar raised ridge across the posterior right side of the opisthosoma. By mirroring the preserved parts of legs 3 and 4 from the right side of the specimen onto the left (Fig. 4b), it can be seen that these structures most likely represent the distal parts of legs 3 and 4. These leg parts appear as raised ridges beneath the opisthosoma, yet the preserved parts of right leg 4 beyond the opisthosoma boundary lies on a level higher in the matrix than the opisthosoma, which at first seems incompatible with such an interpretation. However, if one considers that the carcass was buried in fine volcanic ash, which later became compressed, it is easy to envisage that a leg lying above the opisthosoma would prevent some compaction beneath and so imprint a ghost reflection of its shape as a raised ridge underneath.

## Discussion

It is impossible to know for certain whether the giant male does, indeed, belong to the same species as the previously described female. The situation, however, is akin to the problem of matching sexes in museum collections of extant spiders which have been collected from the same locality but at different times: their co-occurrence in the same horizon and locality, and many similar morphological features, lead to the conclusion of conspecificity. Matching of males and females has proved possible in other geological situations, including the Daohugou beds (Selden and Huang 2010). The similar, large size of both sexes is a striking feature; generally, male spiders are somewhat smaller than females, and extremely so in the case of some Orbiculariae (Vollrath and Parker 1997), and are only very rarely larger (Schütz and Taborsky 2003). Despite several years of intensive fossil collecting at the Daohugou locality, no bigger spider has been discovered than *Mongolarachne*. Many morphological features are shared between the male and female that are not found in other species (mostly undescribed) from Daohugou. While many spiders bear trichobothria in one or two rows on the tibia (where they are commonly short: Forster and Gray 1979), the large cluster of tibial trichobothria found in both sexes of *Mongolarachne* is distinctive. The brushes of long setae (gaiters) occur on all legs in the female of *Mongolarachne* and are also found, though much more weakly, in the male. Other features, such as shorter femoral macrosetae, lack of trichobothria on other podomeres, and plumose setal structure are shared by both sexes.

Phylogenetic placement: The holotype female of *Mongolarachne* was originally placed in the family Nephilidae because features were consistent with the morphology of the extant members of this family, particularly *Nephila* (Selden et al. 2011). The combination of large size, gaiters on the legs, short femoral macrosetae, and lack of femoral trichobothria suggest this genus, although synapomorphies of Nephilidae (there are c. 15, of which about half are behavioral, the rest mostly genitalic: Hormiga et al. 1995) are not visible in the fossil. In contrast, the newly discovered adult male is so unlike modern *Nephila* that a re-evaluation of the placement of the genus is necessary.

**Fig. 5:**
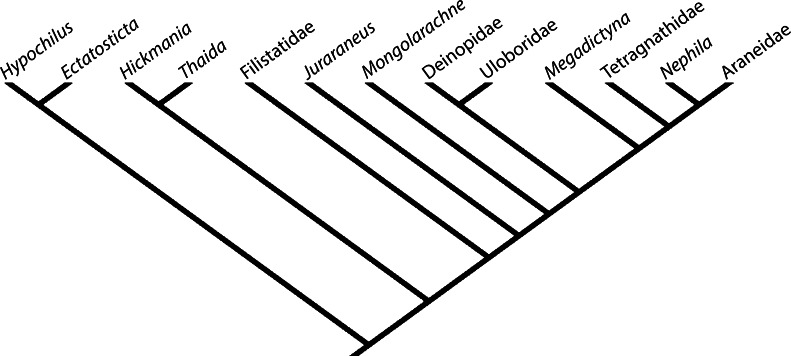
Tree (length 52) derived from Table 1, with modern taxa constrained according to recent hypotheses (e.g., Blackledge et al. 2009; Dimitrov et al. 2012)

It can be seen from the scores in Table 1 that Deinopidae shares the largest number of character states with *Mongolarachne*, followed by *Juraraneus* and *Megadictyna*. Purely out of interest, running Mesquite on the data in Table 1 resulted in 1,978 most-parsimonious trees with a tree length of 47. However, the goal of the present analysis was to try and determine how the fossil taxon relates to the comparators, rather than perform a fruitless phylogenetic analysis on few, disparate taxa and characters. So, a tree was constructed based on recent analyses (e.g., Blackledge et al. 2009; Dimitrov et al. 2012), and *Mongolarachne* was moved around the tree to find the position where the tree length was shortest. A single tree of length 52 (Fig. 5) places *Mongolarachne* as sister to the modern Orbiculariae. Placing Austrochilidae above Filistatidae in the tree (as suggested by some recent hypotheses: Griswold et al. 2005; Agnarsson et al. 2013) produced four trees of length 51 and one of 50 (Electronic supplementary material Fig. 4). The conclusion to be drawn from this analysis is that *Mongolarachne* lies somewhere near the base of the Orbiculariae, perhaps among the cribellate orbicularians, or the stem group to Orbiculariae.

A number of other factors suggest an orbicularian affinity rather than hypochiloid or filistatid. The third leg being particularly short is a feature shared by *Mongolarachne* and most orbicularians, but not the hypochiloids or filistatids. A median tarsal claw and the serrate accessory claws are characteristics of web spiders; ground spiders generally have two tarsal claws, rarely a median claw, commonly specialized claw tufts and/or scopulae, but not serrate accessory claws. The type of web woven by *Mongolarachne* is, of course, unknown; however, the long, thin legs and large body suggest a spider which hangs from a web, either a sheet or vertical web, rather than running on top of a sheet. Among web spiders, tibial gaiters are known only in orbweavers. Their function is poorly known, but crypsis has been suggested for those in *Uloborus* (Opell and Eberhard 1983). In *Nephila*, gaiters appear on legs 1, 2, and 4 in females at about the sixth instar, but some species lose them in adulthood (Robinson and Robinson 1973, 1976). Both males and females of *Mongolarachne* are large, and, while the male’s legs are longer than those of the female, the traditional measurements of body size show the female to be somewhat larger than the male (♀/♂ body lengths 24.67/16.54, ratio 1.49; ♀/♂ carapace widths 6.83/5.05, ratio 1.35; mean ratio 1.42). Among a variety of web spiders, this body size and dimorphism ratio compares closest with *Deinopis* (Deinopidae) and *Eriophora* (Araneidae) (Hormiga et al. 2000). Indeed, sexual size dimorphism is low in most orbicularians, but where extremes occur, e.g., in *Cyrtophora* and *Argiope* (Araneidae), and Nephilidae, the females are very large (Hormiga et al. 2000). The very long pedipalp in the *Mongolarachne* male, especially the elongation of the tibia, is striking. Long pedipalps occur in the males of large, sexually monomorphic spiders, and the unusual elongation of the tibia is especially pronounced in the long-legged hypochilids (Forster et al. 1987), and *Kukulcania* (Filistatidae) (Barrantes and Ramírez 2013), for example. Living hypochiloid spiders weave webs in dark, damp places: beneath rocky outcrops, among boulders, between tree buttresses and roots, in hollow logs, and in caves (Forster et al. 1987). The chances of such a spider becoming trapped in an ash fall in a lake are remote, especially for the sedentary female. So, unless hypochiloids lived in different habitats in the Jurassic, *Mongolarachne* was more likely to have been a weaver of a web in an open habitat close to a lake margin, such as orbicularians today.

Fossil deinopoids (uloborids and deinopids) are known from Cretaceous strata (Dunlop et al. 2013) and undescribed forms also from the Jurassic (pers. obs.: Selden and Huang 2010). Araneoidea are characterized as ecribellate orbweavers, and the loss of the cribellate condition is considered to have occurred before the superfamily emerged within the Orbiculariae. *J. rasnitsyni*, a single adult male from the Middle Jurassic of Transbaikalia (Eskov 1984), was interpreted as an araneoid based on the complexity of the male pedipalp, with a large paracymbium, and the monotypic family Juraranidae was diagnosed on a unique combination of characters found in other araneoid families. Indeed, it has been suggested that *Juraraneus* could be accommodated in Araneidae (Wunderlich 1986). A restudy of the single specimen (Selden 2012) has revealed that it is cribellate, and SEM study (Electronic supplementary material Fig. 2) has shown that this specimen bears plumose setae, albeit different from those in *Mongolarachne*. It has been suggested by M. J. Roberts, (in lit.) that *Juraraneus* is a subadult male, and the structures within the palp are visible but yet to erupt.

## Conclusions

This analysis of the new family Mongolarachnidae, in comparison with Juraraneidae and possible modern relatives, shows that the fossils appear to be related to the orbicularian Deinopidea, and possibly some Nicodamidae and Austrochilidae. This suggests that a greater diversity of cribellate orbicularians existed in the Mesozoic, some survivors of which occur at the present day.

This first use of SEM as a technique for the study of fossil spiders has proved remarkably effective. Indeed, it should now be applied to other, possibly contentious, records of fossil spiders. However, in order to be really practical, it is necessary fully to appreciate the phylogenetic signals of setal ultrastructure among modern spider families. The importance of this was emphasized by Lehtinen (1967, 2013), investigated in a preliminary way by Green (1970), and mentioned by various authors in phylogenetic studies (e.g., Griswold et al. 2005). A new study along the lines of Green’s master’s thesis would be most desirable.

## Electronic supplementary material

Below is the link to the electronic supplementary material.

ESM 1 (PDF 531 kb)
