# Supplementary material for: A giant spider from the Jurassic of China reveals greater diversity of the orbicularian stem group
Source: Naturwissenschaften. 2013 Dec 7;100(12):1171–81. doi: 10.1007/s00114-013-1121-7 (PMC3889289; doi:10.1007/s00114-013-1121-7)
Supplement: Supplementary file 1 — (PDF 531 kb) [file 114_2013_1121_MOESM1_ESM.pdf]

**Electronic Supplementary Materials**

# An enigmatic giant spider from the Jurassic of China

Paul A. Selden[1,2,3], ChungKun Shih[1], and Dong Ren[1]

[1]College of Life Sciences, Capital Normal University, Beijing 100048, China

[2]Paleontological Institute and Department of Geology, University of Kansas, Lawrence, KS 66045, USA

[3]Natural History Museum, London SW7 5BD, UK

## Methods

*Scanning electron microscopy*. Scanning electron microscope photographs were made using an acceleration voltage of either 25kV or 30kV depending on the depth of features examined. Imaging was performed using a 60 μm aperture; detection of electrons was performed by combining 60%/40% mix of electrons collected by a four-quadrant back-scatter electron detector running in composition mode (half of the concentric detector positively charged, and half negatively charged), and secondary electrons were captured using the Everhart-Thornley Detector set a neutral (0) bias. These conditions were used to enhance the contrast of sub-surface compositional differences (<3 μm) in the mineralized materials. Dynamic range was maximized prior to each capture over a new or distinct region of the sample. On occasion, it was necessary to pre-scan an area at a slower dwell time in order to minimize the contributions of charge emission from capacitive regions of the sample, prior to collection of a final image.

*Comparative material*. The following species were examined specifically for comparative purposes for this study [repositories in brackets].

AUSTROCHILIDAE: *Hickmania troglodytes* (Higgins & Petterd, 1883) AUSTRALIA: Tasmania, Weldborough Pass, Rainforest Walk, 28.6 km 280° WNW St Helens, S41.21661°, E147.93852°, elev. 470 m, *Nothofagus* forest. general coll., 6–7 March 2006, C. Griswold & D. Silva OZCG-09 CASENT 9022679 [California Academy, San Francisco]. *Thaida peculiaris* Karsch, 1880, 2♂, 2♀, 8 juv. CHILE: Los Lagos (X): Valdivia: Neltume 23 Nov 1988 V & B Roth coll. det. N. Platnick 1989 CASENT 9021013 [California Academy, San Francisco].

DEINOPIDAE: *Deinopis* sp. ♂: SOUTH AFRICA, Northern Province, Lekgalameetse Reserve, 24°12′S 30°20′E, 18.XII.1995, leg. F. Koch, det. Stefan Otto [Naturkundemuseum, Berlin]. *Deinopis spinosa*? subadult ♂, FLORIDA: Highlands Hammock State Park. 5.IV.1962, coll. M. N. Naumann [University of Kansas Natural History Museum].

NEPHILIDAE: TAIWAN: *Nephila pilipes*; FLORIDA: *N. clavipes*; PANAMA: *N. clavipes*, Barro Colorado Is. [University of Kansas Natural History Museum].

FILISTATIDAE: *Kukulcania hibernalis* (Hentz, 1842). Lake Corpus Christi State Park, Mathis, Texas. 21 March 1982. coll. R. E. Beer. Under eaves of building in typical filistatid web, det. P. A. Selden [University of Kansas Natural History Museum].

HYPOCHILIDAE: *Ectatosticta davidi* Simon, 1888, CHINA, Shaanxi prov. Taibai Shan S flanks, above Houshenzi, mixed conifer/*Rhododendron* forest, 3050m, 14–15 June 1997, P. Jäger, J. + B. Martens leg. (2 tubes, inc. ♂); also same locality, primary broad-leaved forest, 2500–2600m, 25–26 June 1997, J. Martens & P. Jäger leg. (1 tube) [Senckenberg Museum,

Frankfurt] (see {Platnick:2009jn}). *Hypochilus petrunkevitchi* Gertsch, 1958, ♂, det. Platnick 1985. USA: California: Tulane County: Sequoia National Park, 0.9 mile south of Giant Forest Village; elev. 1800m. 17 August 1984. TSBriggs, VFLee, DUbick CASENT 9052229 [California Academy, San Francisco].

NICODAMIDAE: *Megadictyna thilenii* Dahl, 1906. ♂ ♀, det. Joy Boutin 1995. New Zealand, North Island, Wellington. Town belt top of Harriet Street, 24 IV 1995, coll Joy Boutin, CASENT 9052241 [California Academy, San Francisco].

**Supplementary Figures**

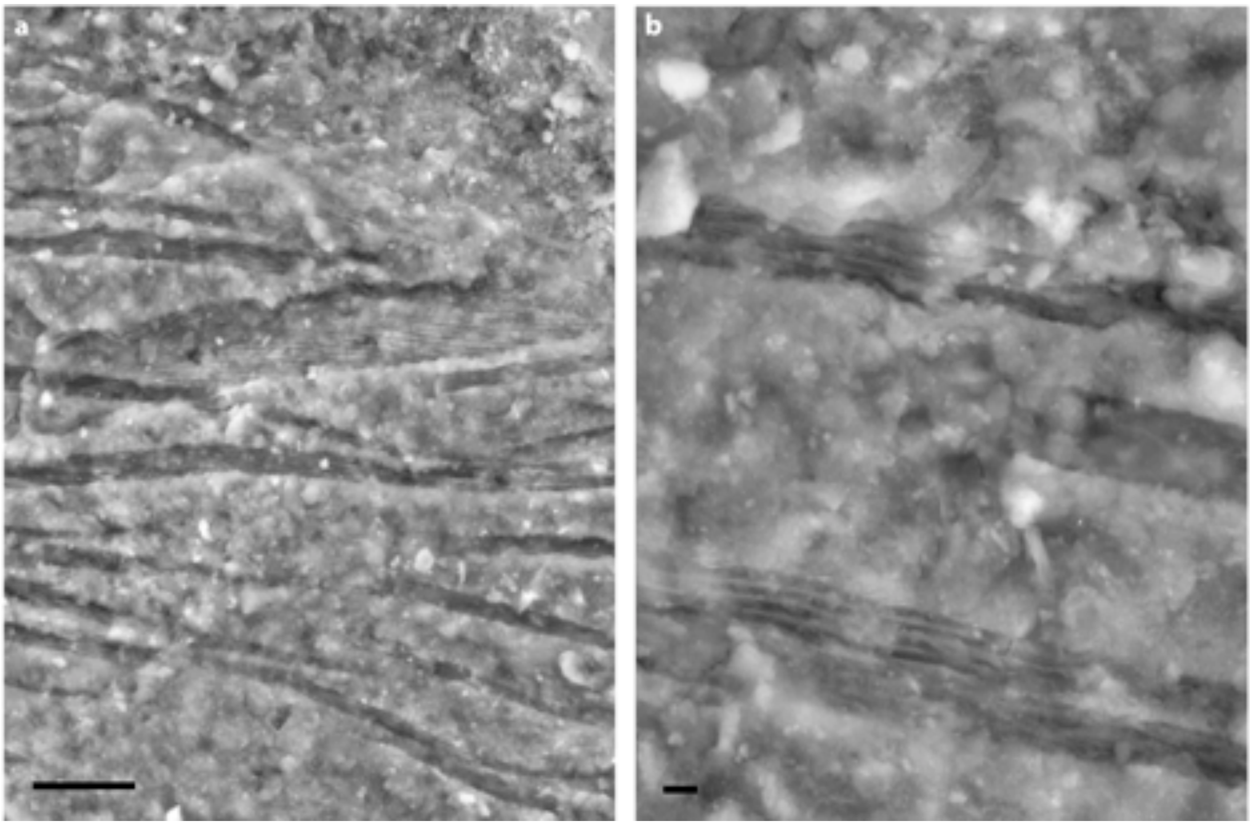

**Supplementary Figure 1.** *Juraraneus rasnitsyni*, holotype male (PIN 3000/3000, Paleontological Institute of the Russian Academy of Sciences, Moscow, from the Upper Jurassic (Oxfordian) Ichetuy Formation of the Tugnuy river valley near Novospasskoye village, Transbaikalia, Siberia): **a** SEM photograph of left leg 4 showing setae and macroseta (centre) with tiny projections; linear pattern on setae and spiral pattern on macroseta; distal to right; scale bar = 20 µm; **b** SEM photograph of part of right leg 1, showing setae with linear pattern and tiny projections; distal to right; scale bar = 2 µm.

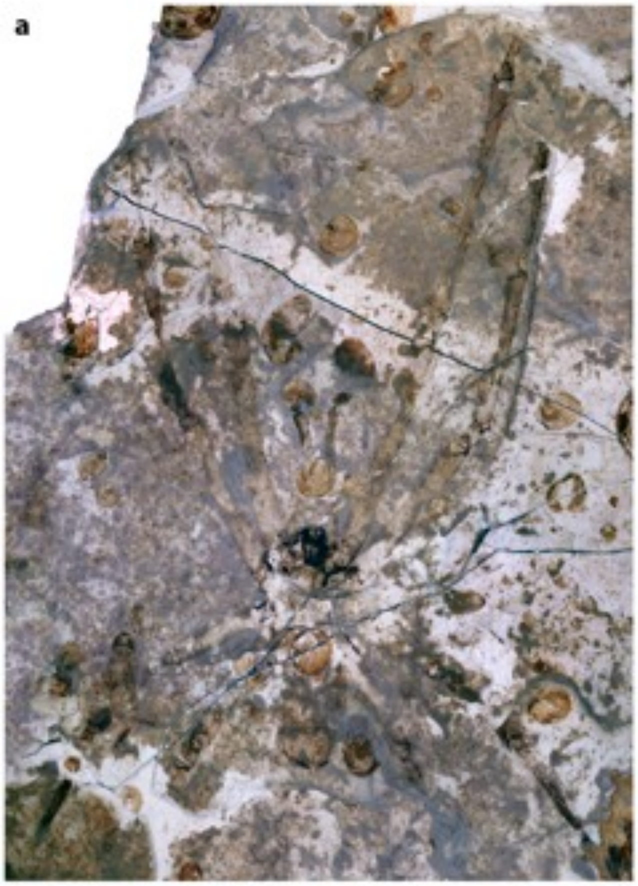


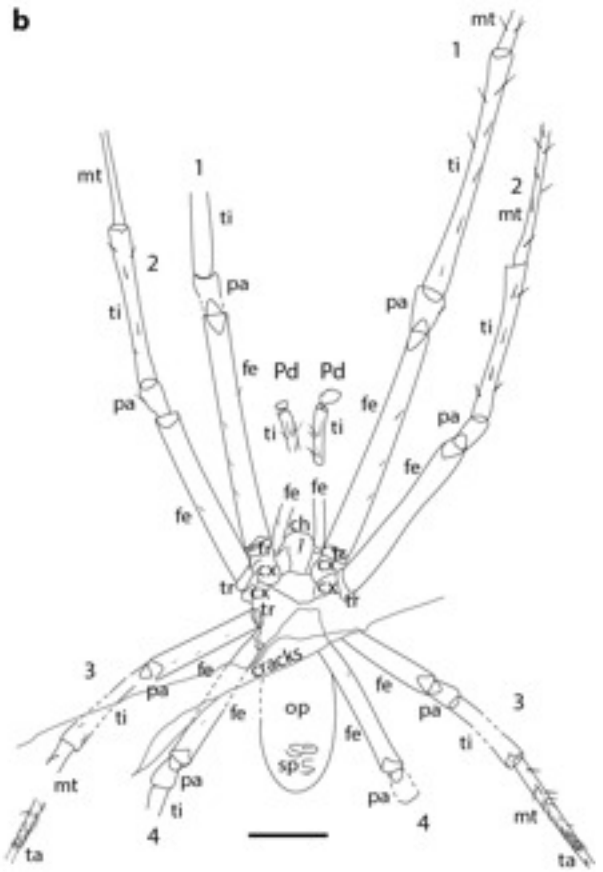


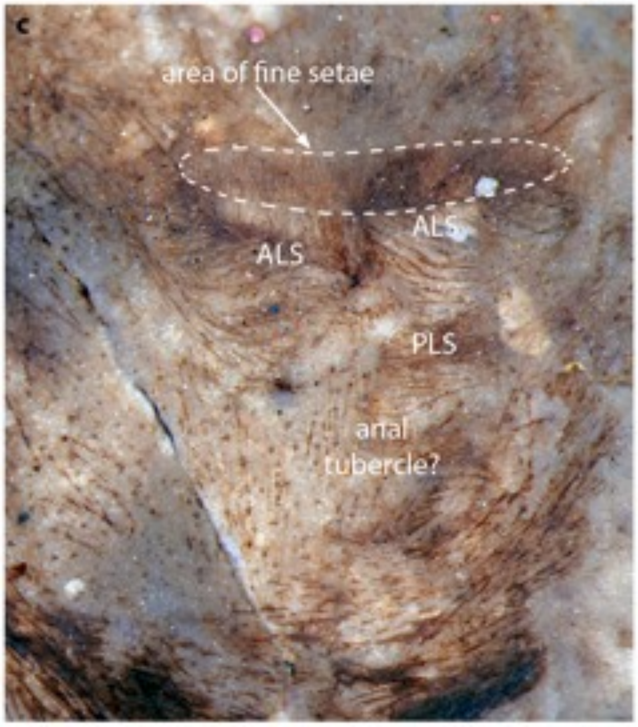


**Supplementary Fig. 2.** *Mongolarachne jurassica*, allotopotype male counterpart (CNU-ARA-NN20110001-2): **a** photograph in under polarized light of specimen in 70% ethanol; see **b** for explanation; **b** Explanatory drawing of **a**; **c** Detail of spinneret region. Abbreviations: 1, 2, 3, 4, leg numbers; ALS, anterior lateral spinneret; ch, chelicera; cr, cribellum; cx, coxa; fe, femur; mt, metatarsus; op, opisthosoma; pa, patella; Pd, pedipalp; PLS, posterior lateral spinneret; sp, spinnerets; ta, tarsus; ti, tibia; scale bar = 5 mm.

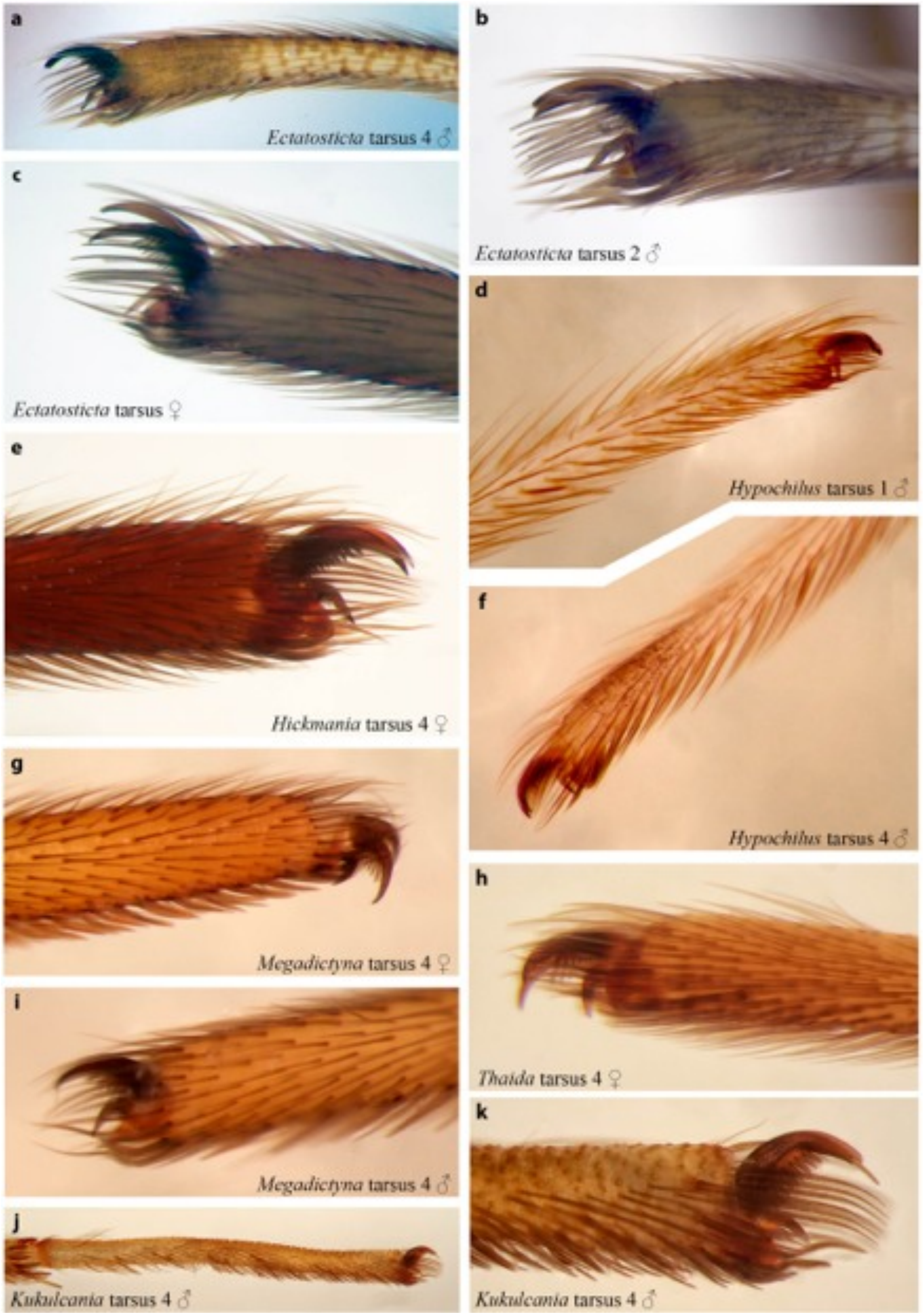


**Supplementary Fig. 3.** Tarsi of comparative spiders.

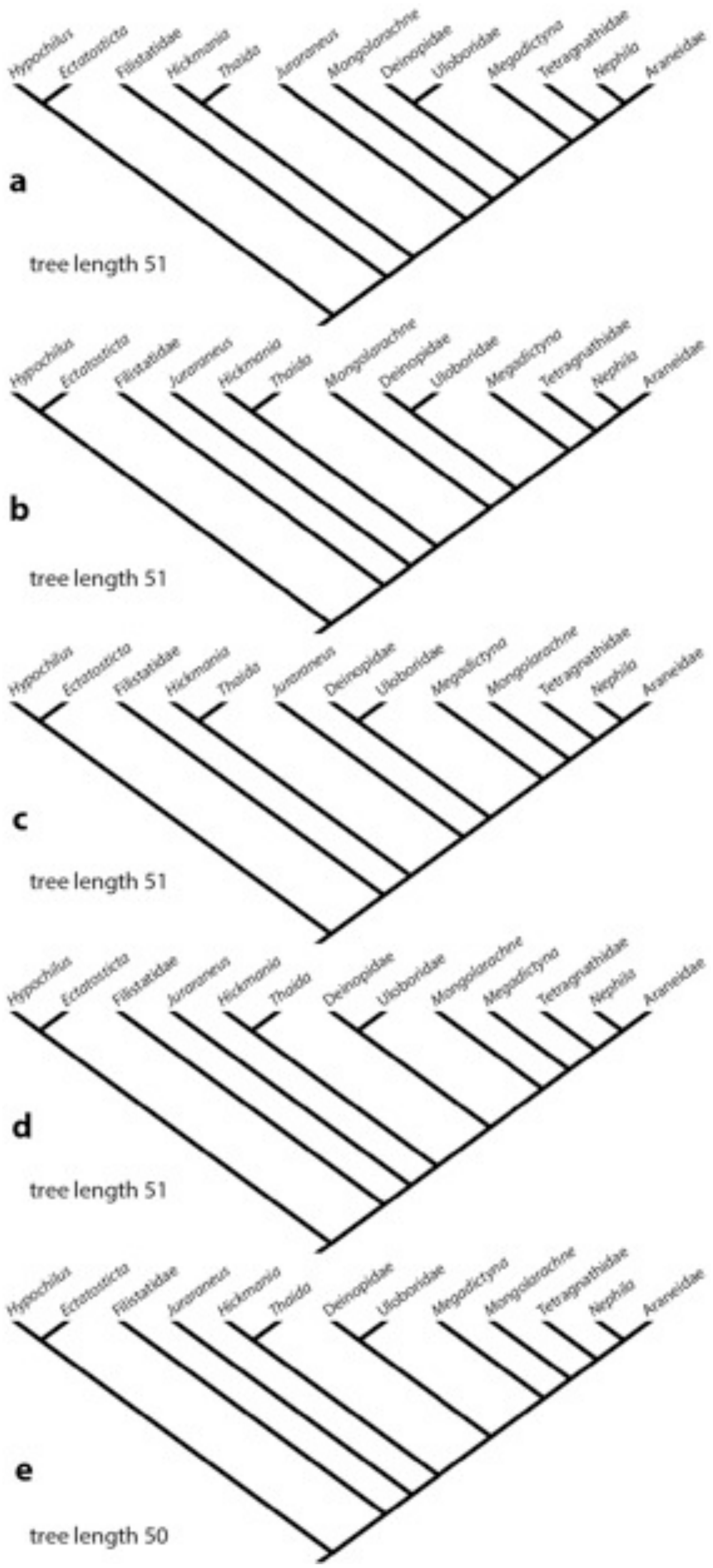


**Supplementary Fig. 4.** Additional trees of length 50 and 51 with Filistatidae between Hypochilidae and Austrochilidae, differing in the placement of *Juraraneus*, and the effect on the hypotheses of relationship of *Mongolarachne*: **a**,**b** *Mongolarachne* sister to Orbiculariae; **c**–**e** *Mongolarachne* nested within Orbiculariae.
